# Negative pressure wound therapy reduces the motility of *Pseudomonas aeruginosa* and enhances wound healing in a rabbit ear biofilm infection model

**DOI:** 10.1007/s10482-018-1045-5

**Published:** 2018-02-21

**Authors:** Wang Guoqi, Li Zhirui, Wang Song, Li Tongtong, Zhang Lihai, Zhang Licheng, Tang Peifu

**Affiliations:** 1 Department of Orthopedics, Chinese PLA General Hospital, No. 28 Fuxing Road, Beijing, 100853 People’s Republic of China; 2 Medical College, Nankai University, Tianjin, 300071 People’s Republic of China; 3 Department of Orthopedics, Tianjin Hospital, No. 406 Jiefangnan Road, Tianjin, 300211 People’s Republic of China

**Keywords:** Negative pressure wound therapy, Infection, Virulence factor, Biofilm, Wound

## Abstract

*Pseudomonas aeruginosa* motility, virulence factors and biofilms are known to be detrimental to wound healing. The efficacy of negative pressure wound therapy (NPWT) against *P. aeruginosa* has been little studied, either in vitro or in vivo. The present study evaluated the effect of negative pressure (NP) on *P. aeruginosa* motility in vitro, and the effect of NPWT on virulence factors and biofilms in vivo. *P. aeruginosa* motility was quantified under different levels of NP (atmospheric pressure, − 75, − 125, − 200 mmHg) using an in vitro model. Swimming, swarming and twitching motility were significantly inhibited by NP (− 125 and − 200 mmHg) compared with atmospheric pressure (p = 0.05). Virulence factors and biofilm components were quantified in NPWT and gauze treated groups using a rabbit ear biofilm model. Biofilm structure was studied with fluorescence microscopy and scanning electron microscopy. Additionally, viable bacterial counts and histological wound healing parameters were measured. Compared with the control, NPWT treatment resulted in a significant reduction in expression of all virulence factors assayed including exotoxin A, rhamnolipid and elastase (p = 0.01). A significant reduction of biofilm components (eDNA) (p = 0.01) was also observed in the NPWT group. The reduction of biofilm matrix was verified by fluorescence- and scanning electron-microscopy. NPWT lead to better histologic parameters (p = 0.01) and decreased bacterial counts (p = 0.05) compared with the control. NPWT treatment was demonstrated to be an effective strategy to reduce virulence factors and biofilm components, which may explain the increased wound healing observed.

## Introduction

Biofilm infections have long been considered to be one of the most difficult problems in wound care (Costerton et al. 1999; Parsek and Singh 2003; Cooper et al. 2014; Akers et al. 2014). A bacterial biofilm can be defined as a complex community of aggregated bacteria embedded within a self-secreted matrix of extracellular polymeric substances (EPS),which include water, polysaccharides, proteins, glycolipids and extracellular DNA (eDNA) (Lindsay and von Holy 2006; Edwards and Harding 2004; Percival and Bowler 2004; Bradley and Cunningham 2013; Li et al. 2015). As a common opportunistic pathogen, *Pseudomonas aeruginosa*, and especially *P. aeruginosa* biofilm-related infections, have been studied widely (Seth et al. 2012b; Watters et al. 2013; Trostrup et al. 2013). Many studies have demonstrated that *P. aeruginosa* biofilms are key factors for aggravating the skin inflammatory response and impairing wound healing (Seth et al. 2012b; Trostrup et al. 2013; Watters et al. 2013). In particular, eDNA is one of the main components of the extracellular matrix of *P. aeruginosa* biofilms, and an association between antibiotic resistance and eDNA has been established (Mulcahy et al. 2008; Chiang et al. 2013). Many virulence determinants of *P. aeruginosa*, including exotoxin A, rhamnolipid and elastase, have been identified. Production of these virulence determinants has been postulated to contribute to the failure of wound healing (Lyczak et al. 2000; Bjarnsholt et al. 2008; Heggers et al. 1992; Schmidtchen et al. 2003). Moreover, initiation of infection and biofilm formation depend on bacterial adhesion within the wound environment, which is facilitated by altered motility and cell surface interactions. *P. aeruginosa* has three types of motility; it can traverse various types of surfaces by swarming, swimming or twitching, depending on the solidity of the environment (Burrows 2012; Kearns 2010; Taguchi et al. 1997). These various modes of surface motility enable bacteria to establish symbiotic and pathogenic associations with plants and animals (Ottemann and Miller 1997).

Various studies have concentrated on the treatment of *P. aeruginosa* biofilm infections of wounds, using various methods to remove biofilms or reduce virulence factor effects. Traditional therapy involving serial debridement and lavage can remove the majority of biofilms, virulence factors and necrotic tissue; however, residual bacteria may rapidly reestablish a robust biofilm architecture, causing pain to the patients during the process (Seth et al. 2012b; Wolcott et al. 2010). Wet-to-dry treatment keeps the wound moist and helps drain wound secretions. However, wet-to-dry treatment is not particularly effective in clearing *P. aeruginosa* from the wound (Lalliss et al. 2010). Although some newly designed dressings for wound care do have an inhibitory effect on biofilms, the efficacy varies greatly between types, concentration and kinetics of the release of active compounds (Seth et al. 2014; Phillips et al. 2015). In addition, some biological agents have been used for targeting bacterial biofilms, such as monoclonal antibodies, although the clinical efficacy and safety of these compounds has not yet been sufficiently evaluated (Kaufmann et al. 2011; Seth et al. 2014).

Negative pressure wound therapy (NPWT) is a medical treatment that has revolutionised the treatment of complex wounds over the last 20 years. For the effective management of contaminated wounds, NPWT has been widely used in clinical laboratories (Armstrong and Lavery 2005; Blum et al. 2012; Seo et al. 2013). NPWT can enhance wound healing despite an infection, although studies have indicated that the bacterial load remained high after NPWT treatment (Lalliss et al. 2010; Yusuf et al. 2013). In a recent systematic review, Glass and colleagues concluded that there was evidence that NPWT exhibits species selectivity, suppressing the proliferation of non-fermentive Gram-negative bacilli including *P. aeruginosa* (Glass et al. 2017). Similar results were found in another study (Liu et al. 2014). This is a possible reason for the accelerated wound healing under NPWT. Most previous studies concentrated on the benefits of the secondary effects of NPWT, including decreased edema, removal of wound exudates, modulation of inflammation and the stimulation of wound healing signaling pathways (Orgill et al. 2009; Streubel et al. 2012). However, investigations regarding the influence of NPWT on biofilms, virulence factors and motility remain limited. Previously, we demonstrated that negative pressure induced by NPWT could change bacteria biofilms and secretion in vitro (Li et al. 2015; Wang et al. 2016). The present study aimed to evaluate the potential effect of NPWT on *P. aeruginosa* motility in vitro, and biofilms and virulence factors in vivo. In addition, bacterial burden and wound healing secondary to NPWT were also investigated.

## Materials and methods

### Animals

All animal experiments were approved by the Medical Ethics Committee of the Chinese PLA General Hospital (Beijing, China). Adult Japanese large-ear white rabbits (aged 3 to 6 months and weighing approximately 3 kg) were acclimated to standard housing and fed ad libitum under constant temperature (22 °C) and humidity (45%) with a 12 h light/dark cycle. A total of 22 rabbits were used to complete this study.

### Bacterial strains and culture

*P. aeruginosa* wild-type strain PAO1 carrying the gene encoding the green fluorescent protein (GFP) was obtained from the laboratory of the Chinese PLA Institute for Disease Control and Prevention (Beijing, China). *P. aeruginosa* was grown overnight at 37 °C and subcultured in Luria–Bertani (LB) broth (AOBOX Biotechnology Co. Ltd., Beijing, China) at 37 °C until log-phase was achieved. Cells were harvested by centrifugation at 4 °C (5000×*g*) and washed three times with phosphate-buffered saline (PBS). Optical density at 600 nm was measured. An optical density of 1.0 was equivalent to 10^5^ colony-forming units (CFU) per microliter, as determined by a standard curve.

### Motility assays in vitro

*P. aeruginosa* motility under different negative pressure (NP) and atmospheric pressure (AP) conditions were evaluated. The bacterial culture protocol was based on our previously published model for the in vitro NP condition and motility medium was prepared as previously described with a few modifications (Rashid and Kornberg 2000; Wang et al. 2016). Briefly, a NP condition was created for bacterial growth and an air tight chamber was used as the incubator. The air was sucked from the chamber by a NP drainage device (WEGO, Weihai, China), which can automatically produce and maintain the NP at − 75, − 125 and − 200 mmHg. The O_2_ concentration was constantly maintained at 20%, as an adequate amount of room air was introduced into the incubator every 5 min.

### Swimming

Tryptone broth (1 g/100 ml tryptone [OXOID], 0.5 g/100 ml NaCl) containing 0.3% agarose (Shanghai Baygene Biotechnology Co. Ltd., China) was used. Swim plates (diameter 35 mm, Corning Life Sciences, USA) were inoculated with bacteria from an overnight culture on LB agar plates at 37 °C using a sterile toothpick. The plates were then wrapped with Saran Wrap to prevent dehydration and inoculated at 30 °C for 24 h.

### Swarming

Media used for swimming assays consisted of 0.5% agar with 0.8 g/100 ml LB broth (1 g/100 ml tryptone, 0.5 g/100 ml yeast extract, 1 g/100 ml NaCl), to which 0.5 g/100 ml glucose was added. Swarm plates were typically allowed to dry at room temperature overnight before being used.

### Twitching

Media used for twitching assays consisted of LB broth solidified with 1% agar. Twitch plates were briefly dried, then strains were stab-inoculated, from an overnight-grown LB agar plate culture, with a sharp toothpick to the bottom of the plates, then incubated at 37 °C for 24 h. Motility plates were randomly assigned to the NP group and the AP group. The zone (maximum diameter) of motility was measured for statistical analysis.

### Biofilm formation in vitro

To quantify biofilm formation, dishes (Corning Life Sciences, USA) with a diameter of 35 mm were used; 2 ml LB broth and 10^6^ CFU *P. aeruginosa* were inoculated into each dish. Dishes were randomly assigned to the NP group and the AP group, then cultured at 37 °C for 24 h without shaking. To stain the biofilm, each dish was washed twice with PBS to remove planktonic cells, then 1 ml 1% crystal violet staining solution was added per dish. Dishes were incubated for 15 min at room temperature, followed by washing three times with PBS. Absorbed stain was eluted from the attached cells on the dishes with 2 ml 95% ethanol and the absorbance was measured by a GeneQuant 1300 spectrophotometer at 595 nm (Li et al. 2015). Similarly, dishes containing only LB broth but no *P. aeruginosa* were used as negative controls.

### Wound protocol and bacteria biofilm model

The wound protocol and bacterial biofilm model was based on a previously published wound model, with minor modifications (Seth et al. 2012b). In brief, rabbits were anesthetised by intramuscular injection with ketamine (45 mg/kg; Gutian Pharma Co., Ltd., Fujian, China) and xylazine (5 mg/kg; Huamu Animal Health Product Co., Ltd., Jilin, China). Ears were shaved, sterilised twice with 70% ethanol and injected with 1% lidocaine (Yimin Pharmaceutical Co., Ltd, Beijing, China) for local anesthesia. Six 8-mm diameter, full thickness, dermal wounds were created on the ventral ear, down to the perichondrium, by surgeons using a scalpel, then dressed with semi-occlusive IV3000 Transparent Adhesive Film Dressing (Smith & Nephew Healthcare Ltd., Hull, UK). All wounds were inoculated with 1 × 10^6^ CFU of *P. aeruginosa* in a volume of 10 µl at postoperative day 3 (POD 3). Bacteria were allowed to proliferate under the semi-occlusive transparent film for 24 h to ensure bacterial biofilm formation (Kanno et al. 2010; Seth et al. 2012b).

### Wound treatment plan

For each animal, the two ears were respectively and randomly assigned to the control group and the NPWT group. A total of 264 wounds from 22 rabbits were used for data analysis.

Clinical treatments were administered to wounds on POD 4. Wounds in the NPWT group were dressed with a standard NPWT dressing (Wuhan VSD Medical Science & Technology Co., Ltd., Wuhan, China) trimmed in advance to the appropriate size. NPWT was set at a pressure of − 125 mmHg throughout the study (Li et al. 2016). Wounds in the control group were dressed with gauze. Dressings were checked daily and changed on PODs 6, 8, 10, 12 and 14. Animals were sacrificed via an overdose of intravenous pentobarbital sodium (100–240 mg/kg; Sigma–Aldrich, St. Louis, MI, USA) on PODs 6 (n = 12), 8 (n = 2), 10 (n = 2), 12 (n = 2) and 14 (n = 4). An 8 mm dermal biopsy punch (Miltex, Inc., York, PA, USA) was used for wound harvesting.

### Detection of virulence factors and eDNA in wound biofilms

The content of exotoxin A, rhamnolipid and elastase secreted by *P. aeruginosa* in each wound was measured at POD 6 to evaluate the early effect of NP on these virulence factors. The dorsal side of the samples were removed to eliminate the possible interference of other bacteria outside of the wound surface. Samples were placed in centrifuge tubes with 1 ml PBS and sonicated to remove bacterial biofilms from the tissue for 2 min. Samples were then centrifuged at 4 °C (13,400×*g*) to remove insoluble substances. The resulting supernatant was used for detection of virulence factors and eDNA.

Exotoxin A was measured according to the method of Shigematsu et al. (2007) using a commercially available Human Pseudomonas Exotoxin A (PEA) ELISA Kit (Cusabio Biotech Co., Ltd., Hubei, PR China), according to the manufacturer’s instructions. The data were recorded as ng/ml.

Rhamnolipid was quantified by the orcinol method, as previously described with a few modifications (Yang et al. 2012). Briefly, 400 μl supernatant was extracted twice using 600 μl diethyl ether. The ether layer was transferred to a fresh tube for evaporation. Residues were dissolved in 150 μl H_2_O, 100 μl 1.6% orcinol (Sigma) and 750 μl 60% sulfuric acid (H_2_SO_4_). After heating for 30 min at 80 °C, the tubes were cooled at room temperature for 30 min and the absorbance at 421 nm was recorded. The concentrations of rhamnolipid were calculated by multiplying rhamnose values by a coefficient of 2.5, as previously described (Pearson et al. 1997). The elastase activity was measured by the elastin-Congo red assay, as previously described (Yang et al. 2012). Briefly, 100 µl supernatant was added to tubes containing 10 mg of elastin-Congo red (Sigma) and 900 µl Na_2_HPO_4_ (pH 7.0). Tubes were incubated for 4 h at 37 °C under shaking conditions and the absorbance at 495 nm was recorded, after removing the precipitate by centrifugation.

Supernatant (300 μl) was used for the detection of eDNA. A TIANamp Micro DNA kit (Tiangen Biotech Co., Ltd., Beijing, China) was used to extract eDNA according to the manufacturer’s protocol (Li et al. 2016). The level per wound of eDNA was expressed as the DNA concentration and data were recorded as μg/ml.

### Viable bacterial counts

Samples were excised as described in the protocol for virulence factors measurement. Tissue samples were homogenised in 1 ml sterile PBS and then sonicated for 2 min to disrupt biofilms. Subsequently, homogenates were serially diluted and plated on *P. aeruginosa* Isolation Agar plates (Sigma) and incubated overnight at 37 °C. Standard colony counting methods were used to determine the number of CFU (Seth et al. 2012b; Li et al. 2016).

### Scanning electron microscopy

Wound samples were fixed in 2.5% glutaraldehyde and 1% osmium tetroxide fixation successively. Samples were dehydrated using an ethanol series and then were dried with a critical point dryer (HCP-2; Hitachi, Ltd., Tokyo, Japan) by flooding with liquid carbon dioxide at 5 °C for 20 min and raising the temperature to the critical point. Subsequently, samples were mounted by means of double-sided tape and coated with gold in an auto sputter coater (E-1010; Hitachi, Ltd., Tokyo, Japan). Samples were visualised using a scanning electron microscope (S-3400 N; Hitachi, Ltd., Tokyo, Japan) operated at the scanning voltage of 15 kV.

### Imaging aggregates in wound sections

Each wound was divided into two equal parts and embedded in O.C.T. compound (Sakura Finetek USA, Inc., Torrance, CA, USA), quickly frozen and then tissue sections were obtained with a Leica CM1950 freezing microtome (Leica Microsystems GmbH, Wetzlar, Germany). *P. aeruginosa* glycocalyx was visualised by staining tissue sections with 150 µg/ml of Concanavalin A, Alexa Fluor™ 647 Conjugate (Invitrogen; Thermo Fisher Scientific, Inc.) for 15 min in the dark at room temperature. The sections were then washed 3 times with PBS and incubated with DAPI (4′,6′-diamidino-2-phenylindole dilactate, Invitrogen; Thermo Fisher Scientific, Inc.) to visualise the host cells (Watters et al. 2013; Kanno et al. 2010). An Olympus BX51 microscope (Olympus Corporation, Tokyo, Japan) was used to visualise different fluorescence.

### Wound closure measurement

Images of the wounds were captured with a digital camera (IXUSi; Canon, Inc., Tokyo, Japan) at the dressing changing point from POD 0. Image-Pro Plus version 6.0 software (Media Cybernetics, Inc., Rockville, MD, USA) was used to determine the wound size quantitatively. The rate of wound healing was expressed as a percentage of the initial wound area (Li et al. 2016).

### Histological analysis

Wounds were bisected at their largest diameter and fixed in 10% neutral formalin. Samples were then embedded in paraffin, cut into 5 µm sections and stained for analysis. An Olympus MVX10 macro-microscope was used to observe slides. Image-Pro Plus version 6.0 software (Media Cybernetics, Inc., Rockville, MD, USA) was used to quantify of epithelial and granulation gaps and total epithelial or granulation area (Seth et al. 2012a; Li et al. 2016). Two independent observers, who were blinded to the treatment, carried out measurements and completed the calculation of the average results.

### Statistical analysis

Data are presented in graphic form as the mean ± standard deviation when applicable. Student’s t-test (2-tailed and paired) was used when comparing 2 study groups including virulence factors, eDNA, viable bacterial counts and histological parameters. Significant changes in motility colony diameters were determined using the one-way ANOVA test. Repeated-measures analysis of variance, followed by the least significant difference post-hoc test, was used to analyse wound closure. The level of significance was set at p < 0.05. * indicates significant differences (p < 0.05), ** indicates very significant differences (p < 0.01) and *** indicates highly significant differences (p < 0.001).

## Results

### Negative pressure resulted in reduced motility and biofilm formation in vitro

**Fig. 1:**
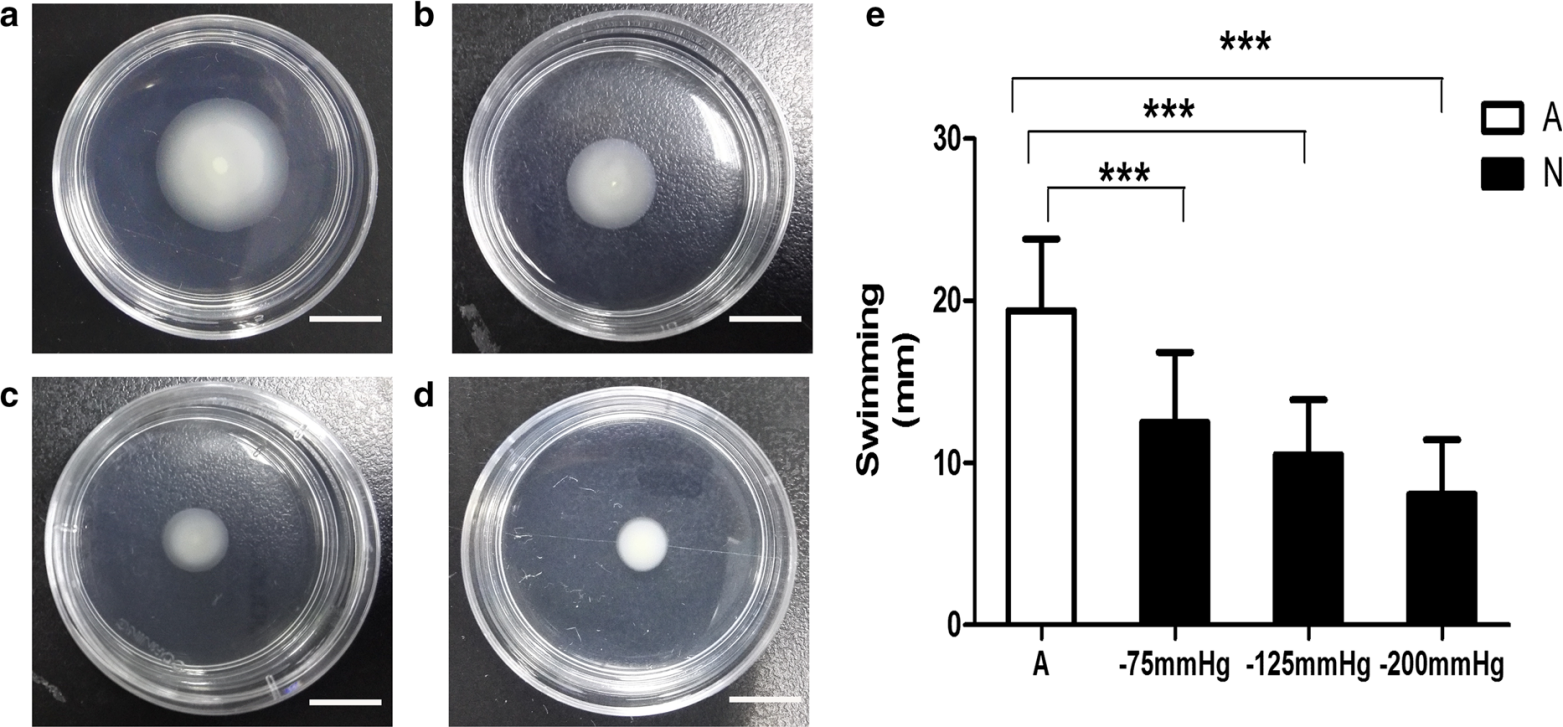
Swimming motility of *P. aeruginosa* cells pretreated with and without negative pressure in vitro. **a**–**d** Swim colonies of *P. aeruginosa* cells inoculated onto swim plates exposed to atmospheric pressure, − 75 mmHg, − 125 mmHg, or − 200 mmHg for 24 h. **e** Mean diameters of swim colonies (mm) are presented as mean ± standard deviation (N = 12). All swim colonies produced by negative pressure-treated cells were significantly smaller than those under atmospheric pressure. (p < 0.001). A = atmospheric pressure, N = negative pressure; Scale bar = 10 mm

**Fig. 2:**
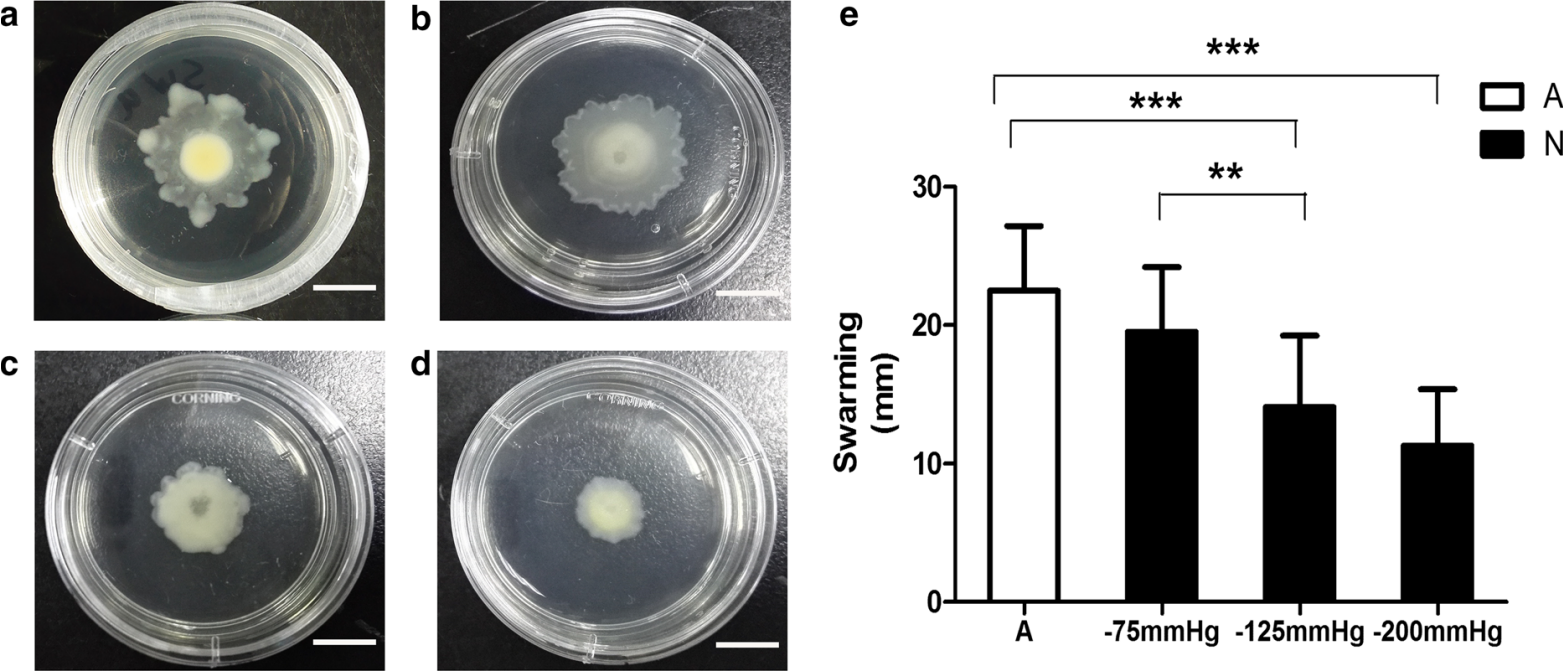
Swarming motility of *P. aeruginosa* cells pretreated with and without negative pressure in vitro. **a**–**d** Swarm colonies of *P. aeruginosa* cells inoculated onto swarm plates exposed to atmospheric pressure, − 75 mmHg, − 125 or − 200 mmHg for 24 h. **e** Mean diameters of swim colonies (mm) are presented as mean ± standard deviation (N = 12). Swarm colonies produced by negative pressure-treated (− 125 or − 200 mmHg) cells were significantly smaller than those under atmospheric pressure. (p < 0.001). A = atmospheric pressure, N = negative pressure; Scale bar = 10 mm

**Fig. 3:**
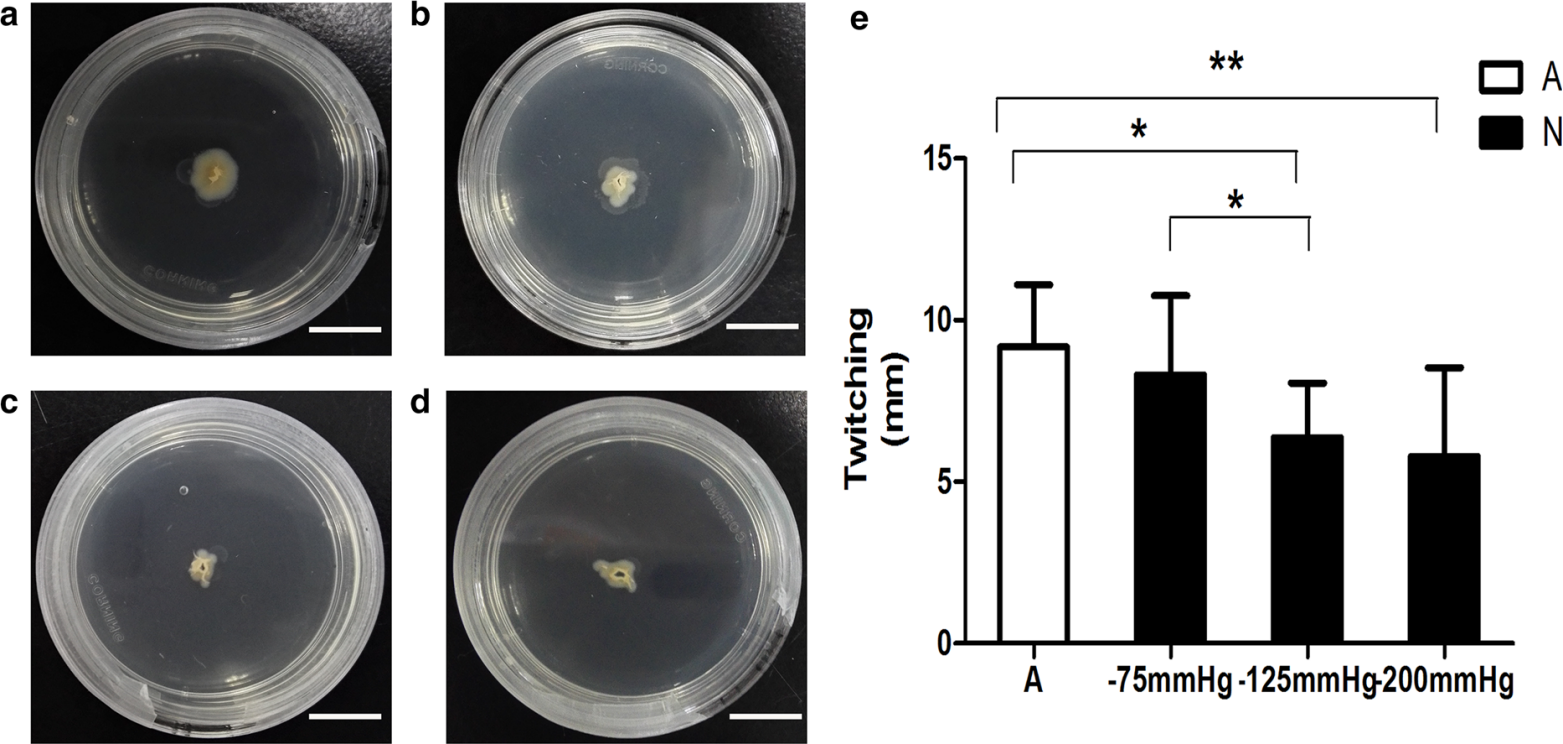
Twitching motility of *P. aeruginosa* cells pretreated with and without negative pressure in vitro. **a**–**d** Twitch colonies of *P. aeruginosa* cells inoculated onto twitch plates exposed to atmospheric pressure, − 75, − 125 or − 200 mmHg for 24 h. **e** Mean diameters of twitch colonies (mm) are presented as mean ± standard deviation (N = 12). Twitch colonies produced by negative pressure-treated (− 125 or − 200 mmHg) cells were significantly smaller than those under atmospheric pressure. (− 125 mmHg p < 0.05; − 200 mmHg p < 0.01). A = atmospheric pressure, N = negative pressure; Scale bar = 10 mm

Under different pressures used, there was a pressure-dependent reduction in motility (assessed as swimming, swarming and twiching) in response to NP treatment (Figs. 1–3). Movement through the agar was significantly impeded as the pressure decreased, especially at − 125 and − 200 mmHg, compared to the control. However, movement was not completely abrogated under any pressure. Swimming was retarded under − 75 mmHg compared with the control (Fig. 1). However, this was not observed for swarming and twitching (Fig. 2, 3).

**Fig. 4:**
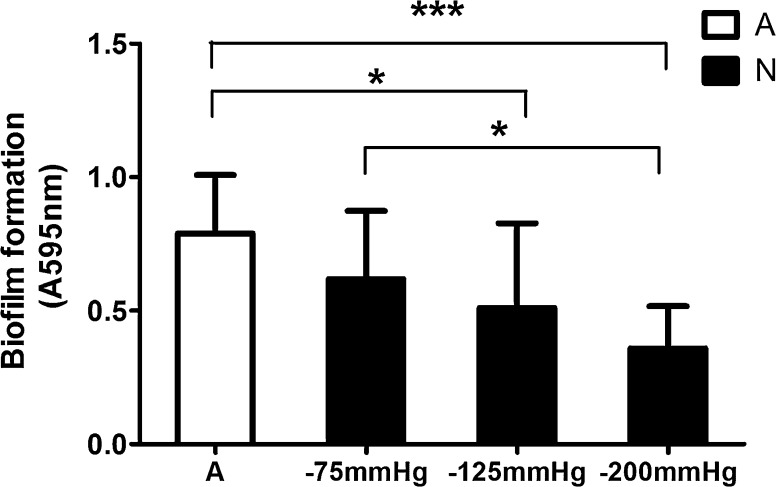
Biofilm formation of *P. aeruginosa* cells pretreated with and without negative pressure in vitro. Crystal violet staining of biofilms was quantified at an absorbance of 595 nm (N = 12). Negative pressure (− 125 or − 200 mmHg) showed an inhibitory effect on biofilm formation compared with the control (− 125 mmHg p < 0.05; − 200 mmHg p < 0.001). A = atmospheric pressure, N = negative pressure

Biofilm formation measured in vitro indicated that NP (− 125 and − 200 mmHg) lead to a significant decrease in biofilm formation compared with the control (Fig. 4, p < 0.05 and p < 0.001, respectively).

### Detection of virulence factors and eDNA in wound biofilms

**Fig. 5:**
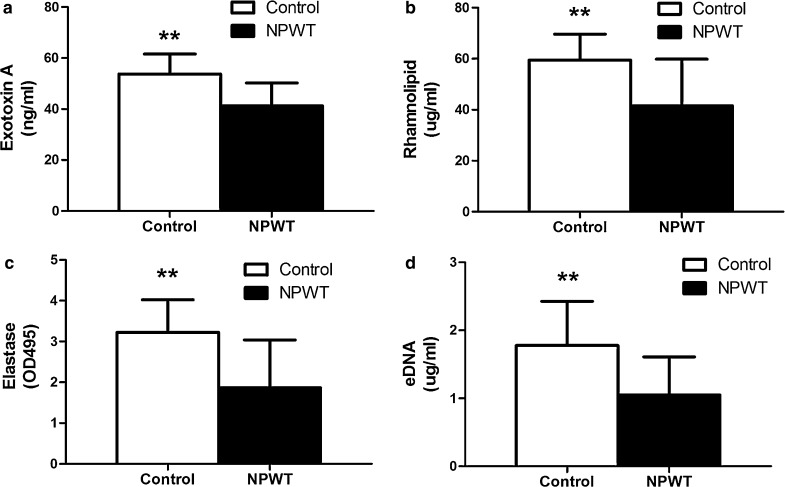
Detection of virulence factors and components of the biofilm matrix from *P. aeruginosa*-infected wounds with and without NPWT treatment at POD 6 (in vivo). NPWT resulted in significant reductions in **a** Exotoxin A, **b** Rhamnolipid, **c** Elastase and **d** eDNA content relative to the controls (p < 0.01). Data are presented as mean ± standard deviation (n = 10–12 wounds/group). NPWT, negative pressure wound therapy; eDNA, extracellular DNA

The three virulence factors detected in the NPWT group were significantly reduced compared to the levels in the control group (Fig. 5a–c, p < 0.01). For eDNA, one of the main components of the *P. aeruginosa* biofilm, NPWT resulted in a significantly lower level in the wound beds (Fig. 5d, p < 0.01).

### Viable bacterial counts

**Fig. 6:**
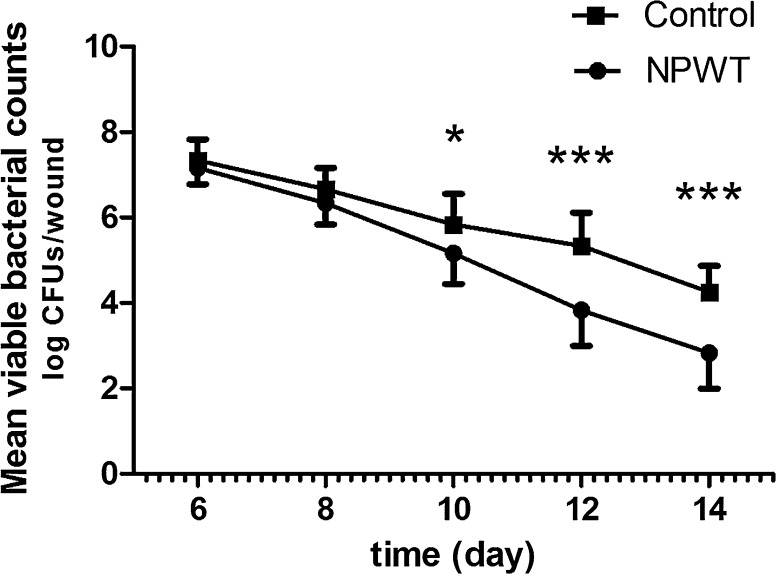
Viable bacterial counts from *P. aeruginosa*-infected wounds treated with NPWT and control (in vivo). Bacterial counts from the controls indicated a persistent bacterial burden averaging between 10^4^–10^7^ CFUs/wound. However, wounds treated with NPWT showed a gradual but significant reduction in viable bacteria compared with controls, with ~ 10^3^ CFUs/wound on POD 14. *P < 0.05, **P < 0.01 and ***P < 0.001. Data are presented as mean ± standard deviation (n = 10–12 wounds/group/time-point). NPWT, negative pressure wound therapy; CFUs, colony-forming units; POD, postoperative day

Bacterial load in the two groups was estimated through measuring the viable bacterial counts at different times (Fig. 6). Bacterial burden in the NPWT group was not significantly different compared to the control on POD 6 and 8, although a reduced bacterial burden was observed which became a significant reduction in bacterial count at POD 10, 12 and 14 compared with the control (POD 10, p < 0.05, POD 12 and 14, p < 0.001). Moreover, in a preliminary experiment, we have assessed gentamicin (IM, 1 mg/Kg per 8 h) as a positive control. However, bacteria were quickly killed and could not be detected when systemic antibiotic was administered (data not shown). Thus, antibiotic treatment is more effective than NPWT treatment in this model and so we did not add an antibiotic treatment group.

### Imaging aggregates in wound sections and scanning electron microscopy

**Fig. 7:**
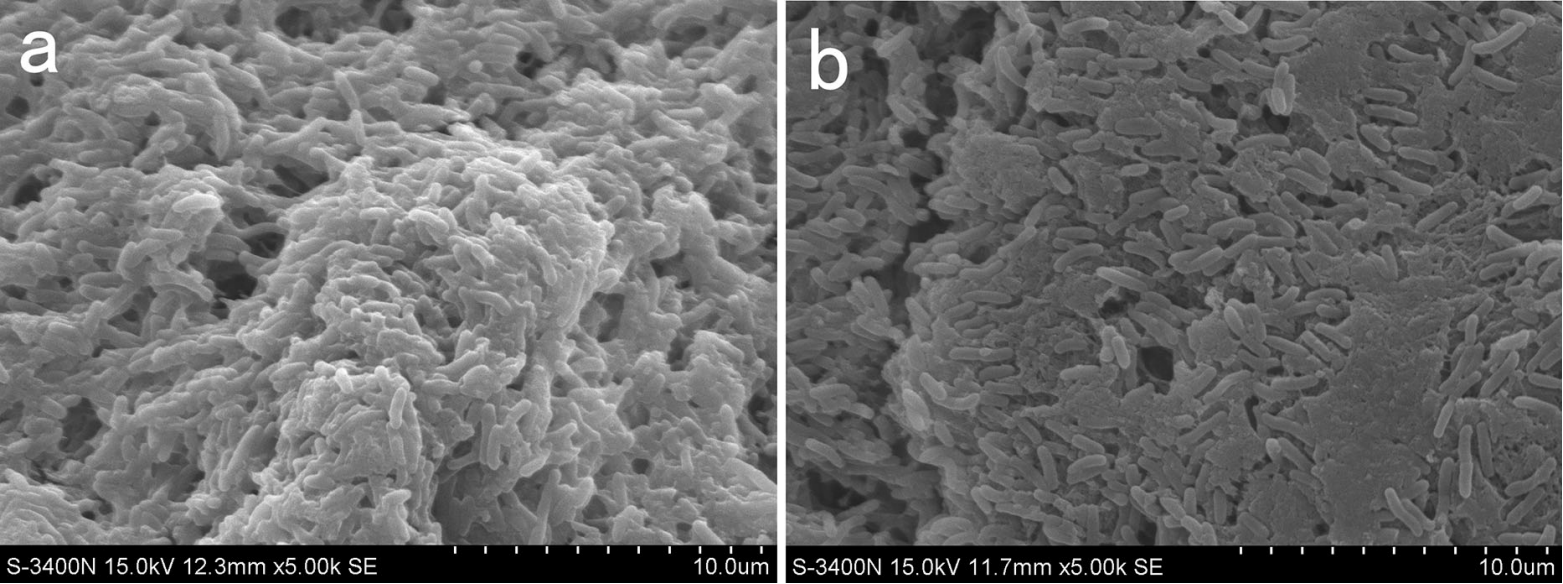
Scanning electron microscopic images of *P. aeruginosa* biofilm–infected wounds with NPWT and control at POD 6 (in vivo). **a** Control wounds exhibited a mature biofilm structure. **b** Wounds treated with NPWT presented numerous individual rod-shaped bacteria and lack of extracellular matrix. NPWT, negative pressure wound therapy

**Fig. 8:**
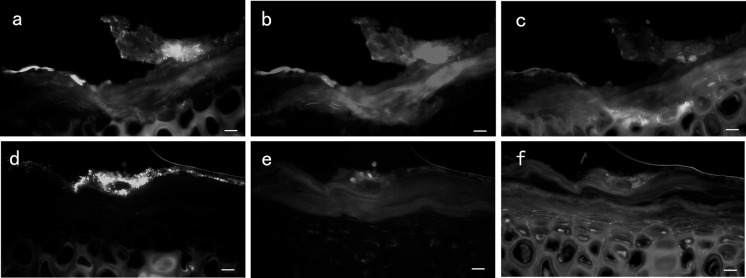
Fluorescence light microscopy of NPWT and control treated *P. aeruginosa*-infected wounds counterstained with ConA and DAPI at POD 6 (in vivo). **a**–**c** GFP-labeled *P. aeruginosa* (green) formed a mature biofilm on the control wound surface (blue), showing a complex structure with large amounts of glycocalyx (red) around the bacteria. **d**–**f** GFP-labeled *P. aeruginosa* (green) in the NPWT group spread over the wound bed (blue) with sparse glycocalyx matrix, although large aggregates of *P. aeruginosa* are visible. Scale bar = 100 µm. NPWT, negative pressure wound therapy; ConA, Concanavalin A; DAPI, 4’,6-diamidino-2-phenylindole; GFP, green fluorescent protein. (Color figure online)

The presence of biofilms in wounds is known to be a key impeder in wound healing for many reasons, including providing protection from endogenous and exogenous antimicrobial agents, stimulating a chronic state of inflammation, and impeding re-epithelisation through a mechanical barrier (James et al. 2008; Romling and Balsalobre 2012). However, few of these theories have been tested in vivo under NPWT treatment. Wounds in the control group showed relatively large aggregates, with considerable extracellular matrix and intact biofilm structure (Fig. 7a). Conversely, wounds treated with NPWT manifested smaller aggregates and a lack of extracellular matrix (Fig. 7b). Bacteria in wounds in the NPWT group spread over the wound beds, with regional aggregation and sparse glycocalyx, although aggregates were still large (Fig. 8d–f). However, large amounts of glycocalyx surrounding the bacteria were observed in the control wounds (Fig. 8a–c).

### Wound closure measurement and histological analysis

**Fig. 9:**
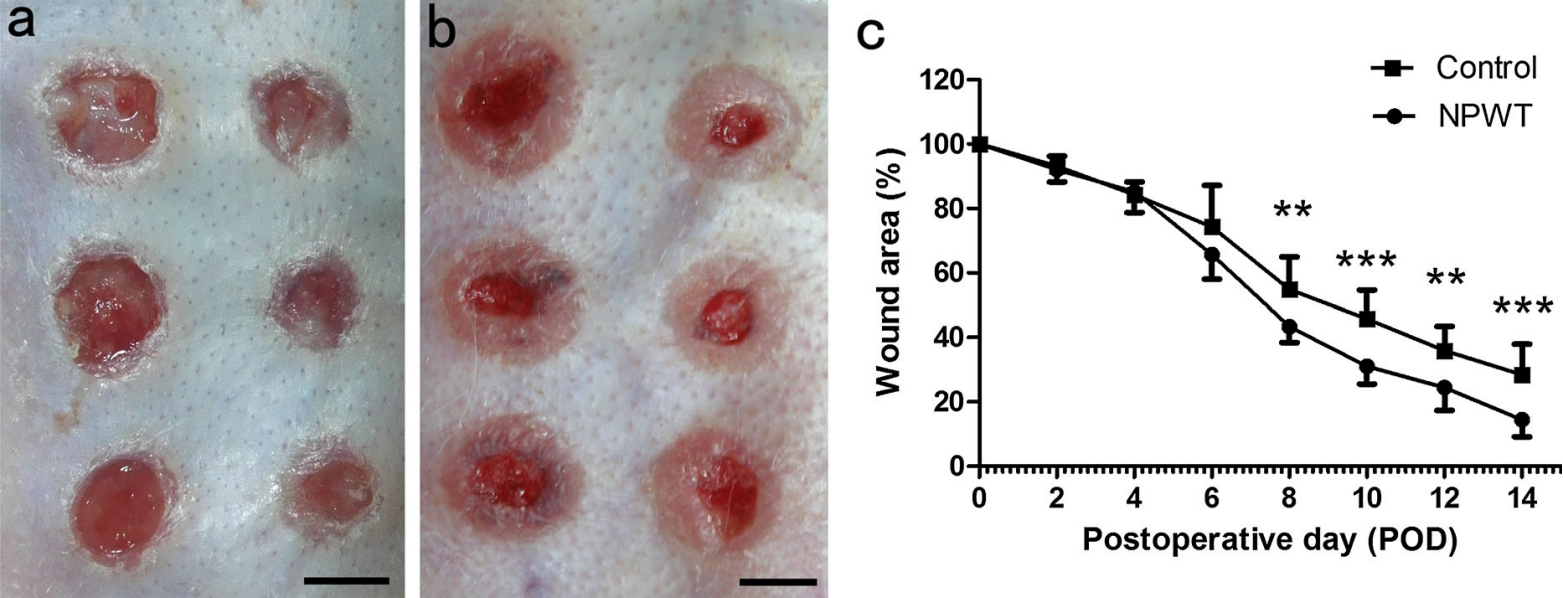
Appearance of wounds and wound closure rates. **a** Wounds under control conditions manifested a delay in healing. **b** NPWT accelerated wound closure and epithelialisation, with clean granulation tissue beds at POD 14. **c** The wound closure rate, shown as the percentage of initial wound area, significantly increased in the NPWT group compared with the control group from POD 8. (**a** and **b**) Scale bar = 5 mm. *P < 0.05, **P < 0.01 and ***P < 0.001. Data are presented as mean ± standard deviation (n = 12 wounds/group). NPWT, negative pressure wound therapy; POD, postoperative day

**Fig. 10:**
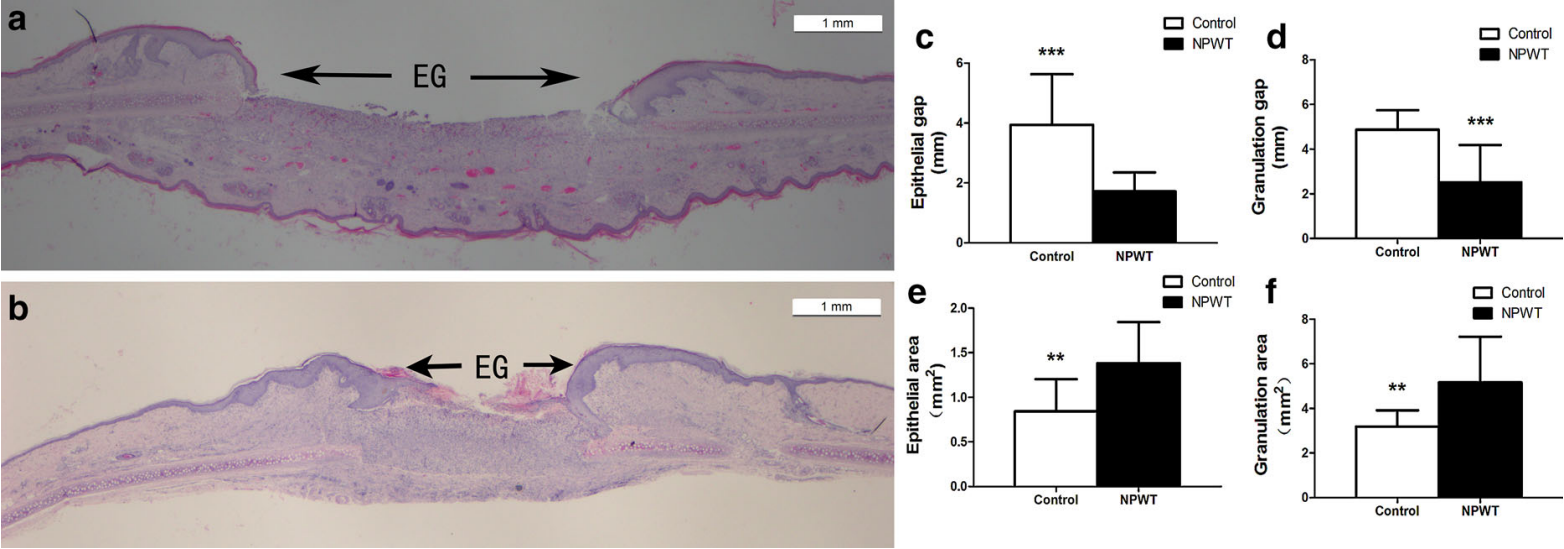
Histologic comparison of *P. aeruginosa* biofilm–infected wounds following NPWT and control treatments. Images of wounds at POD 14 showed that the amounts of new epithelial and granulation tissue in the NPWT-treated wounds **b** were significantly increased compared to the controls **a**. As determined by quantitative analysis **c** and **d**, NPWT was shown to result in improvements in all healing parameters, with a significant reduction in epithelial and granulation gaps, and increase in new epithelial and granulation areas **e** and **f**. Scale bar = 1 mm. **P < 0.01 and ***P < 0.001. Data are presented as mean ± standard deviation (n = 10–12 wounds/group). EG, epithelial gap; NPWT, negative pressure wound therapy; POD, postoperative day

To verify that the reduction of bacterial burden secondary to NPWT treatment was accompanied by an enhancement in wound healing, the wound closure, epithelia and granulation were measured quantitatively. The wound closure area in the two groups decreased gradually as the treatment progressed. The rate of wound closure in the NPWT group was significantly higher than in the control (Fig. 9a–c) after POD 8. Histological analysis demonstrated that NPWT treatment lead to a significant (p < 0.001) improvement in the epithelial and granulation gap (Fig. 10a–d, epithelial gap, p < 0.001; granulation gap, p < 0.001) as well as increasing the new epithelial and granulation tissue area (Fig. 10a, b, e, f, epithelial area, p < 0.01; granulation area, p < 0.01) compared with the control, as observed through quantitative measurement of these histological parameters.

## Discussion

NPWT has been utilised as a ubiquitous wound management resource and is widely used for acute open wounds, burn wounds, and chronic wounds, as well as infected wounds (Stannard et al. 2009; Kantak et al. 2017; Chiang et al. 2017; Lo Torto et al. 2017). The goals of our study were to utilise an in vitro model and bacteria biofilm model to determine whether NPWT inhibits *P. aeruginosa* motility, reduces biofilm formation and virulence factors in wounds, and to assess if these effects counteract impaired wound healing. *P. aeruginosa* swarming and swimming are both flagellum-dependent, whereas twitching depends on type IV pili. The potential benefits of motility include ability to access to optimal colonisation sites, translocate to preferred hosts, as well as dispersal in the environment, during the course of an infection. In vitro results indicated that the application of NP may be an effective approach for suppression of motility and biofilm formation. Swimming, swarming, and twitching were all retarded under − 125 mmHg pressure or lower, but no level of NP tested in this study inhibited motility completely. However, reduced motility may help impede further dispersal in wounds. Similarly, biofilm formation was decreased under NP, especially at − 200 mmHg, but still not completely. A pressure of − 200 mmHg appears to be decrease motility and biofilm formation most effectively. However, − 200 mmHg is not the most often recommended pressure clinically, because NPWT at this pressure would increase pain in wounds. Biofilms were observed in wounds inoculated with *P. aeruginosa* PAO1 carrying the gene encoding GFP. A previous study found that topical NP compressed the biofilm architecture, with a reduction in thickness and diffusion distance (Ngo et al. 2012). Unlike the study of Ngo et al. (2012), the present investigation predominantly focused on some components of biofilms. We observed that biofilms were more prevalent in the control wounds, and NPWT lead to sparse bacterial glycocalyx and extracellular matrix, without any spread/dispersal. Detection of another key constituent of biofilms, eDNA, further supported our hypothesis that NPWT could block the formation and persistence of bacterial biofilms in vivo to some extent, but not completely eradicate established biofilms. This may be due to the drainage and negative pressure of NPWT, as previous studies have verified that NPWT is an effective method for removal of extracellular fluid and wound cleaning due to the suction (Lancerotto et al. 2012).

Without protection from the biofilm matrix, the bacteria may be more exposed to the host immune cells and therefore be more easily eliminated (Boles and Horswill 2011), which is consistent with the viable bacterial counts observed here. A previous study reported that bacteria counts under NPWT increased or decreased, but still exceeded 10^5^ CFU/g tissue. In this study, bacteria counts were high in both groups on POD 6 and 8, and no significant difference was observed. However, bacterial counts in the NPWT treatment group were lower than 10^5^ CFU/per wound after POD 12. Reduction of virulence factor levels under NPWT treatment was another likely benefit to host tissue, as virulence factor activities can lead to tissue necrosis. These results may partly explain the observed significant differences in wound closure, epithelial and granulation tissue area between NPWT treatment and the control, although NPWT itself could accelerate wound closure. Underlying these improvements in wound healing is the ability of NPWT treatment to disrupt bacterial biofilms, which is consistent with the growing consensus that biofilms are critical in delaying keratinocyte migration and wound granulation (James et al. 2008; Schierle et al. 2009; Watters et al. 2013).

There are limitations to our study. Firstly, motility under NP was estimated only in vitro. Little information was gathered in vivo due to methodological difficulties. Secondly, we limited our study to a single bacterial species, *P. aeruginosa*. As the majority of patients have mixed infections, future studies regarding other bacterial biofilm infections will be used to validate the results presented here. Additionally, NPWT combined with other therapies, such as irrigation or anti-biofilm agents, was not used. NPWT alone could only inhibit the formation and persistence of biofilms but could not eradicate them completely, demonstrating the durability of biofilms and indicating the need for persistent and aggressive therapy. For future studies, combined treatments will be performed to investigate the efficiency of bacterial biofilm removal.

*P. aeruginosa* biofilm infections are prevalent in clinical patients. It is important to recognise the commitment required to perform effective clinical wound care for these patients. In this study, NPWT was demonstrated to be a relatively effective therapy to inhibit *P. aeruginosa* motility, biofilm formation in vitro, and to reduce virulence factor levels in vivo. In particular, NPWT appears to reduce the biofilm matrix, including glycocalyx and eDNA, which may provide opportunities for wound healing. A better understanding of NPWT for infected wounds may help doctors complete effective management of wound care.
